# Society Meetings

**Published:** 1907-12

**Authors:** 


### SOCIETY MEETINGS.

The sixteenth annual meeting of the Association of Military Surgeons was held at the Jamestown Exposition, Norfolk, Va., October 15-18, 1907, and was one of the most interesting in the history of the association.

The Enno Sander prize medal was won by Major Charles Lynch, U. S. A., who is well-known in Buffalo having served for a time as surgeon at Fort Porter. The second prize, a life membership in the association, was awarded Major Henry I. Raymond, who also was stationed at Fort Porter, for a time subsequent to the Spanish war. The subject of the competition was: "What is the most effective organisation of the American National Red Cross for war and what should be its relations with the medical departments of the army and navy?" Dr. Lynch's paper is published in the current issue of the Military Surgeon and is a most able essay.

The public meeting was held under the chairmanship of Surgeon Charles Poindexter Wertenbaker, P.H. and M.H.S. The response to the address of welcome was made by Lieutenant Colonel A. H. Briggs, N.G.N.Y., of Buffalo, after which Major James Evelyn Pilcher, secretary of the association and editor of the *Military Surgeon*, installed the foreign delegates as corresponding members and decorated them with the beautiful insignia of the association. His address of welcome to the delegates was brimming with felicitations and scintillating with wit. These delegates were decorated: Major Carlos Amezcua, Mexico; Colonel Ramon Bengoechea, Guatamala; Colonel Ho Shou-jen, Expectant Prefect, Chinese army; Lieutenant-Colonel William B. Leishman, Royal Army medical corps; Lieutenant-Colonel Murray Maclaren, Canadian A.M.S; Major Arthur Henry Moorehead, Indian medical service; Major Lorenzo Bonomo, Italian army.

The papers read were of practical value and covered all phases of military medicine. Among the most interesting were: "A practical portable *x*-ray equipment for army field-service," by Major John A. Metzger, U.S.V.; "The effects of the sun and of artificial heat," Dr. Harold D. Corbusier, late U.S.A.; "The phychic phenomena of intestinal toxemias and their treatment," by Captain J. Carlisle DeVries, N.G.N.Y.; "The conquests of preventive medicine," by Major Louis L. Seaman; "Tumor of the brain simulating gumma," by Captain Louis T. Hess, U.S.A.

A resolution was passed calling upon congress to pass the army medical reorganisation bill and pointing out the dangers of delay. The following resolution, concerning the acting assistant surgeons and the contract surgeons was unanimously passed:

WHEREAS, the Acting Assistant Surgeons and Contract Surgeons who have served in the United States Army, although they were engaged in the same services, endured the same privations and hardships and suffered the same diseases and injuries as the enlisted and commissioned forces of the United States Army, have no legal standing as such.

*Resolved*, that this Association recommends to Congress that in simple justice legislation be enacted to authorise the President of the United States to commission every Acting Assistant Surgeon or Contract Surgeon who served during the Spanish-American War or the subsequent Philippine hostilities, as a First Lieutenant in the United States Army and muster him out of the service the following day.

Major Pilcher made an eloquent plea for recognition of the contract surgeons, and called attention to the fact that these men by reason of their unofficial positions were ineligible for membership in the majority of patriotic societies.

The election of officers resulted as follows: President, Asst. Surg. Gen. George Tully Vaughan, P.H., and M.H.S., Washington, D.C.; first vice president, Rear Admiral

Presley M. Rixey, U.S.N., Washington, D.C.; second vice-president, Colonel Joseph K. Weaver, N.G. Pa., Norristown, Pennsylvania; third vice president, Colonel William C. Gorgas, U.S.A., Ancon, Canal Zone, Panama; secretary, Major James Evelyn Pilcher, U.S.V., Carlisle, Pennsylvania; treasurer, Major Herbert A. Arnold, N.G. Pa., Ardmore, Pennsylvania; assistant secretary, Captain J. Carlisle DeVries, N.G.N.Y., The Glen Springs, Watkins Glen, N.Y.

Advisory board, Hon. V. H. Metcalf, secretary of navy; Hon. G. B. Cortelyou, secretary of treasury; Rear Admiral P. M. Rixey, U.S.N.; Hon. Wm. H. Taft, secretary of war; Gen. Robert M. O'Reilly, U.S.A.; Gen. Walter Wyman, P.H. and M.H.S.

Executive council, Med. Dir. Manley H. Simons, U.S.N.; Major Jefferson R. Kean, U.S.A.; Lt. Col. Homer I. Jones, Ind. N.G.; Captain Myles Standish, M.V.M.; Major Thomas C. Clark, Minn. N.G.; Asst. Surg. Gen. John W. Kerr, P.H. and M.H.S.; in conjunction with the ex-presidents, ex-officio, viz.: Col. Nicholas Senn, Ill. N.G.; Brig. Gen. J. D. Griffith, N.G. Mo.; Brig. Gen. A. J. Stone, Minn. N. G.; Maj. Gen. Robert Allen Blood, M.V.M.; Lt. Col. Albert H. Briggs, N.G.N.Y.; Brig. Gen. Geo. M. Sternberg, U.S.A.; Colonel John Van R. Hoff, U.S.A.; Medical Director John C. Wise, U.S.N.; Surg. Gen. Walter Wyman, P.H. and M.H.S.; Colonel Valery Havard, U.S.A.

The announcement of the appointment of the following committees closed the business of the association.

Literary committee, Major W. Fitzhugh Carter, U.S.A., Fort Monroe, Va.; Capt Jesse Rowe, I.N.G.; Captain J. Carlisle DeVries, N.G.N.Y.; Surg. William Hemphill Bell, U.S.N.; Dr. Nelson W. Wilson, U.S.A.; Passed Asst. Surg. Wm. C. Rucker, P.H. and M.H.S. Publication committee, Major Jamse Evelyn Pilcher, U.S.V., Carlisle, P.; A. A. Surg. Wm. H. Marsh, P.H. and M.H.S. Lieut. Col. Charles Adams, Illinois, N.G. Necrology committee, Major Samuel Cecil Stanton, Ill. N.G., 1040 Sheridan Road, Chicago, Ill.; Surgeon F. L. Pleadwell, U.S.N.; Lieut. David Munson Trecartin, C.N.G. Public service medical school committee, Surgeon Charles P. Wertenbaker, P.H. and M.H.S., Norfolk, Va.; Major Charles Lynch, U.S.A.; Med. Insp. Henry G. Beyer, U.S.N.; Major Buell S. Rogers, I.N.G. Committee on legislation, Major Trevanian V. Dupuy, O.N.G., Irontown, Ohio; Lt. Col. Julius F. Henkel, Mich. N.G.; Med. Insp. Samuel H. Dickson, U.S.N.; Major Junius F. Lynch, Va. Vols.; Lt. Col. Nathan S. Jarvis, N.G.N.Y.; Brig. Gen. William H. Devine, M.V.M.; Col. Henry Bacon, Fla. N.G.; Lt. Col. Wilbur S. Watson, Conn. N.G.; Major David S. Fairchild, Iowa, N.G.; Major W. D. McCaw, U.S.A.; Major Henry Allers, N.G.N.J.

The social side of the meeting was most enjoyable. There were receptions almost without number; trips to Fort Monroe, trips on the revenue cutter, Pimlico, and a visit to the man-o-war, Brooklyn.

---

THE Southern Surgical and Gynecological Association will hold its next annual meeting at the St. Charles Hotel, New Orleans, December 17, 18, and 19, 1907, under the presidency of Dr. Howard A. Kelly, of Baltimore. Dr. W. D. Haggard, of Nashville, is the secretary and Dr. Denegre Martin, of New Orleans, is the chairman of the committee of arrangements. It is announced that the railroads have declared a return rate of one and one-third fare on the certificate plan. A special rate has been arranged with the proprietors of the St. Charles Hotel.

---

THE Buffalo Academy of Medicine held meetings during the months of October and November as follows:

*Section on Surgery.*—Tuesday evening, October 1. Program: (*a*) Report of case of cerebral tumor simulating gumma and exhibits of blood specimens of various types of malaria found in the Philippines, Captain L. T. Hess, M. D., Fort Porter; (*b*) Traumatic paralysis of the third nerve, with report of case, Lee Masten Francis; (*c*) Intestinal obstruction, with report of cases, J. B. Young.

*Section on Pathology.*—A Stated Meeting was held Tuesday evening, October 15. Program (*a*) The viability of the typhoid bacillus under normal conditions, H. D. Pease, Albany, N. Y.; (*b*) Multiple epitheliomatosis; report of case, A. E. Woehnert.

*Section on Obstetrics and Gynecology.*—Tuesday evening, October 22. Program: (*a*) Lithopedion,—with report of cases, Herman E. Hayd; (*b*) Our friend the enemy, T. H. McKee.

*Section on Medicine.*—Tuesday evening, October 29. Program: Complications of Typhoid, DeLancey Rochester.

*Section on Surgery.*—Wednesday evening, November 6. Program: Report of case of foreign body removed from the bladder with operating cystoscope, David E. Wheeler; (*a*) facial-hypoglossal anastomosis, with illustrations on the cadaver, Geo. F. Cott; (*b*) vesicular diseases, Nelson W. Wilson.

*Section on Medicine.*—Tuesday evening, November 12 . Program: Care of infant during first week of life, Dewitt H. Sherman; recurrent empyema, J. H. Pryor.

*Section on Pathology.*—Tuesday evening, November 19. Program: The shape and relations of the stomach; demonstrations of specimens, Professor A. T. Kerr, Professor of Anatomy, Medical Dept., Cornell University, Ithaca, N. Y.

